# Understanding the Personalities of Patients Who Sustained Minor Injuries Attending the Emergency Department in Hospital Universiti Sains Malaysia

**DOI:** 10.21315/mjms-12-2024-943

**Published:** 2025-04-30

**Authors:** Bing Chan Shu, Nik Hisamuddin Nik Abdul Rahman, Syaheedatul Iman Dinsuhaimi

**Affiliations:** 1 Department of Emergency Medicine, School of Medical Sciences, Universiti Sains Malaysia, Health Campus, Kelantan, Malaysia; 2 Hospital Pakar Universiti Sains Malaysia, Universiti Sains Malaysia, Kelantan, Malaysia; 3 Department of Psychiatry, School of Medical Sciences, Universiti Sains Malaysia, Kelantan, Malaysia

**Keywords:** personality inventory, accidents, psychosocial intervention, risk behaviours, injury prevention

## Abstract

**Background:**

Injuries from road traffic accidents, falls, and other causes are a global health burden. In Malaysia, while mechanical and geographical factors in injuries are well studied, the role of psychosocial aspects, such as personality traits, remains underexplored. This study investigates the influence of personality traits, measured by the Universiti Sains Malaysia Personality Inventory (USMaP-i), on minor injury occurrence among patients at Hospital Universiti Sains Malaysia, focusing on extraversion, neuroticism, openness, agreeableness, and conscientiousness.

**Methods:**

A cross-sectional study was conducted with 150 adult patients, comparing those with minor injuries to a control group without injuries. The Big Five personality traits were assessed using the USMaP-i, and associations with injury risk were analysed using independent *t*-tests, Pearson’s chi-square tests, and multiple logistic regression (MLR).

**Results:**

Significant associations were identified between specific personality traits and injury risk. Higher levels of extraversion were correlated with an increased injury risk, whereas openness demonstrated a protective effect. Gender also played a role, with males showing a 2.8- fold higher likelihood of injury than females. Other traits, such as neuroticism, agreeableness, and conscientiousness, were not significantly associated with injury occurrence.

**Conclusion:**

Extraversion and openness significantly influenced the injury risk. The findings of this study enable the development of evidence-based prevention strategies through i) personality-based screening in the emergency department to identify high-risk individuals, particularly those with elevated extraversion scores; ii) targeted safety education programmes addressing trait-specific risk behaviours; and iii) gender-specific interventions focusing on male risk-taking tendencies. These tailored approaches can enhance existing injury prevention frameworks by incorporating psychological and behavioural factors alongside traditional safety measures.

## Introduction

Injuries from road traffic accidents, falls, acts of violence, burns, drowning, and poisoning contribute to a substantial global health burden, with an estimated 4.4 million injury-related deaths annually, particularly affecting low-and middle-income countries (1). Road traffic injuries (RTIs) are a leading cause, accounting for approximately 1.35 million deaths each year and leaving another 30–50 million individuals with non-fatal injuries that often result in long-term disability (2). In Malaysia, RTIs rank as the second highest contributor to disability-adjusted life years (DALYs) lost, underscoring the urgent need for comprehensive injury prevention strategies (3). While significant emphasis has been placed on mechanical, legislative, and environmental factors, there has been limited focus on psychosocial elements, particularly personality traits, which are increasingly recognised as influencing injury risk behaviours (2, 4).

Personality traits—stable predispositions influencing behaviour over the long term—may play a crucial role in determining individual injury risk. However, little research has addressed this association within the context of Malaysia (5). Global studies show that personality factors, particularly those identified in the Big Five model—extraversion, neuroticism, openness, agreeableness, and conscientiousness—are significantly associated with risk-taking behaviours that impact injury susceptibility (6–7). Extraversion has been linked to increased risk behaviours such as speeding and thrill-seeking, while conscientiousness promotes caution and adherence to safety guidelines (8–9). Additionally, early theories of “accident proneness” by Farmer and Chambers (1926) as cited in Burnham (2008) suggest that some individuals are inherently more likely to experience accidents due to stable personality characteristics (10).

Despite these findings, there is a significant research gap in understanding the role of personality traits in injury risk among the Malaysian population. Local studies have primarily focused on demographic, geographic, and mechanical factors, with a limited examination of how personality traits influence injury-related behaviours. Addressing this gap is essential for developing targeted, population-specific injury prevention strategies that consider psychosocial influences alongside traditional safety measures. This study aimed to assess the relationship between the Big Five personality traits and minor injury occurrence among patients in a Malaysian hospital setting, utilising the USM Personality Inventory (USMaP-i). By exploring personality factors as potential predictors of injury risk, the research seeks to contribute to a more holistic, population-targeted approach to injury prevention, potentially enhancing the effectiveness of safety programmes in Malaysia by integrating behavioural and psychosocial dimensions (5).

## Methods

### Data Collection

This cross-sectional study was conducted from January 2023 to December 2024 at Hospital Universiti Sains Malaysia (USM) in Kubang Kerian, Kelantan, focusing on adult patients aged 18 years old and above who visited the emergency department (ED) with minor injuries. The study was limited to the green zone of the ED, where patients with less severe conditions were treated. The target population included individuals who sustained minor injuries across Kelantan, while the source population specifically involved those who sought treatment at Hospital USM during the study period. Patients were classified into two groups, namely, a study group of individuals with minor injuries and a control group of non-injury-related illnesses.

### Inclusion and Exclusion Criteria

For the study group, the inclusion criteria required stable adults over 18 years old who sustained minor injuries, defined by an Injury Severity Score (ISS) of less than nine. Exclusions included those with a pain score above 3, in distress, with traumatic brain injuries, illiterate, altered sensorium (Glasgow Coma Scale (GCS) < 15), or mental disorders, such as dementia. Elderly patients over 60 were also excluded due to concerns regarding the USMaP-i applicability. The control group included adults aged more than 18 years old with non-injury-related conditions (e.g., fever and cough). The exclusion criteria were similar to those of the study group, ensuring comparable demographics.

### Research Tool

The study used the USMaP-i, a validated questionnaire that assesses the Big Five personality traits of extraversion, neuroticism, openness, agreeableness, and conscientiousness. The questionnaire, containing 60 items and six “faking-good” items, demonstrated internal consistency, with Cronbach’s alpha values ranging from 0.634 to 0.831. Administered in Bahasa Malaysia, the inventory required informed consent from participants before inclusion.

### Statistical Analysis

Categorical data were presented as frequencies and percentages, and numerical data were expressed as means and standard deviations (SD). Simple logistic regression (SLR) was used to identify variables associated with minor injuries, with variables showing a *P*-value below 0.25 included in a multiple logistic regression (MLR) analysis. The MLR used forward, backward, and stepwise methods to identify the most parsimonious model, with the final model including variables with *P*-values < 0.05. Statistical analyses were performed using the Statistical Package for the Social Sciences (SPSS) version 28 (IBM Corp., Armonk, NY, US).

## Results

The statistical analyses used in this study included Pearson’s chi-square tests for categorical variables and independent *t*-tests for continuous variables to examine differences between patients with minor injuries and those without injuries. Logistic regression analyses (simple and multiple) were also conducted to determine the associations between personality traits and injury risk. In the preliminary SLR analysis, a higher *P*-value threshold of 0.25 was used to account for the small sample size, helping to ensure that important variables were not excluded too early. This approach reduces the risk of missing genuine associations in exploratory studies (11).

Of the 150 participants, 60% were men and 40% were women. Table 1 illustrates gender-based differences, with males showing a notably higher injury rate (*P* < 0.001). Other demographic factors, such as age and education level, were not significantly associated with the likelihood of injury. Personality traits have been linked to injury risk. As shown in Table 2, individuals with higher extraversion scores exhibited an increased injury risk (*P* = 0.033), whereas openness had a protective effect, with lower scores observed in the injured group (*P* = 0.023). No statistically significant associations were identified between neuroticism, agreeableness, or conscientiousness.

Logistic regression analysis, as detailed in Table 3, confirmed gender, extraversion, and openness as significant predictors of injury. Males were 2.80 times more likely to experience injuries than females (*P* < 0.004), while higher extroversion increased the odds of injury [odd ratio (OR) = 1.092, *P* = 0.012], and openness reduced it (OR = 0.916, *P* = 0.011). Further validation with MLR, as shown in Table 4, upheld these associations. Table 5 compares personality traits between genders, revealing that females scored higher in openness, indicative of greater creativity and curiosity (*P* = 0.029), whereas other traits showed no significant gender differences.

## Discussion

Globally, injuries from road accidents, falls, burns, drowning, and poisoning result in 1.35 million deaths and 30–50 million non-fatal injuries each year(1). Research increasingly shows that personality traits impact injury-related outcomes, influencing recovery, rehabilitation, and behavioural responses (12). By understanding these traits, healthcare providers can develop more effective and personalised prevention and intervention strategies. This study explored the relationship between personality traits and minor injuries among patients in the ED at Hospital USM, with findings indicating significant associations between specific traits and injury occurrence, along with notable gender differences.

### Gender and Injury Risk

The findings demonstrated a strong association between gender and injury occurrence, with males showing a markedly higher injury rate than females. This finding supports previous research suggesting that males, due to sociocultural factors and a tendency toward riskier behaviours, may have a higher propensity for injuries (13). Understanding this correlation could be valuable for designing targeted interventions to reduce injury risk among males, particularly by promoting risk awareness and safer behavioural practices (14–15). Additionally, males are twice as likely to experience crashes and three times more likely to report multiple incidents (16). However, it is essential to avoid overgeneralising. At the same time, males may generally take more risks, and individual differences within each gender exist, influenced by sociocultural factors and personality traits such as aggression and risk tolerance (17).

### Age and Injury Risk

Although gender emerged as a significant predictor of injury occurrence, no statistically significant association was found between age groups and injury rates, contrasting with previous findings. Research has shown that younger females tend to be safer drivers than males, though this trend reverses in older age groups, with middle-aged drivers generally experiencing fewer accidents (18). The absence of age-related differences in the present study may be due to a sample primarily under the age of 40 years old, potentially obscuring patterns typically seen in older, frailer individuals who are more vulnerable to severe injuries, even from minor traumas (19). Examining a wider range of injury severities in future research could help clarify age-related risk factors across diverse populations.

### Personality Traits and Injury Proneness

This study identified extraversion and openness as key personality traits associated with injury risk. Individuals with higher extraversion scores appear more susceptible to risk-taking behaviours, which may increase their injury risk, whereas those with higher openness scores demonstrate more cautious tendencies.

#### Extraversion and Injury Risk

Extraversion was positively associated with injury risk, as higher scores for this trait correlated with increased susceptibility to risky behaviours. This finding is consistent with prior studies showing that extroverted individuals, characterised by sociability and a preference for excitement, often engage in behaviours that heighten injury risk, such as aggressive driving and rule-breaking (20–21). Research also associates extraversion with aggressive driving, especially among young males (6), with a risk-taking propensity, hostility, and aggression playing roles in predicting traffic offense (22). Extraversion has even been linked to increased physical injury risk in hospital patients and athletes, emphasising the trait’s broad influence on behaviour and safety (23, 24).

#### Openness and Injury Prevention

The present study suggests that openness may be protective against such injuries. Research suggests that higher openness can promote sensitivity and tolerance, potentially reducing injury risk (25). Moreover, individuals with high openness have been shown to exhibit adaptive stress responses, aiding calm decision-making under pressure (26). However, the relationship with driving behaviour is complex. For example, teens high in openness were found to be more likely to engage in distracted driving (27). This mixed evidence suggests that openness generally acts as a protective factor but may vary by context and specific behaviours, necessitating further research (28).

#### Neuroticism, Conscientiousness, and Agreeableness

This study did not find significant associations between injury risk and neuroticism, conscientiousness, or agreeableness scores. This outcome contrasts with some previous research that links neuroticism to increased injury risk due to stress reactivity (29) and conscientiousness to safer behaviours (7). However, the lack of significant findings here may be due to differences in study design, population, or cultural factors. Further research should explore how these traits influence injury risk in other contexts and populations.

### Gender Differences in Personality Traits

The findings align with other research showing that females generally exhibit higher levels of openness than males, a trait associated with creativity, curiosity, and safer driving behaviours, which may potentially reduce injury risk (21, 30). This study also found no significant gender differences in conscientiousness, agreeableness, or neuroticism, supporting previous research that suggests openness is a key differentiator in driving behaviour. Conscientious and agreeable individuals of both genders tend to display safer habits, while neuroticism is associated with riskier driving (21, 31). A cross-cultural study across 55 countries found that women tend to report higher neuroticism, agreeableness, and conscientiousness, which may contribute to more cautious behaviour and lower injury risk; however, higher neuroticism could also increase vulnerability due to stress (32).

The present study’s findings advocate a tailored injury prevention approach that considers gender and personality traits. Interventions for females should focus on strengthening traits such as conscientiousness and openness to further reduce injury risks. Conversely, programmes that address impulsivity and encourage mindful risk assessment may be especially beneficial for males. Such personalised interventions can be integrated into broader public health strategies to create more effective injury prevention programmes.

### Implications for Injury Risk Prediction

This research identified extraversion and openness as key personality traits linked to injury risk, suggesting that personality factors may help predict injury susceptibility. Individuals with high extraversion and low openness scores are more likely to engage in risky behaviours, such as dangerous driving, which increases their injury risk. Males generally score higher on extraversion and lower on openness, showing a stronger association with injury involvement. However, it is crucial to avoid overgeneralising personality traits based solely on gender; conscientiousness and agreeableness, for example, show minimal gender differences, indicating the need for tailored, individualised treatment approaches (33).

Healthcare providers should also account for cultural and age-related variations in personality traits, as these factors influence behaviour and injury risk across populations (34). Ethical considerations, such as avoiding stereotypes and ensuring data privacy, are essential when using personality traits for risk prediction. Personality assessments should focus on supportive interventions rather than restrictive labels, allowing healthcare and educational systems to integrate these insights into injury prevention strategies.

### Practical Implications

Understanding the link between personality traits and minor injuries can enhance patient management and outcomes. Recognising that individuals with high extraversion scores are prone to riskier behaviours allows healthcare providers to offer post-injury tailored counselling on safety and responsible conduct. For example, injury prevention programmes in New Zealand provide behaviour modification counselling for risk-prone personalities, such as extroverts (35). Public health campaigns targeting extroverts with specific safety measures can also address personality-driven risk factors, while interventions for those with lower openness scores can promote caution and mindfulness.

Beyond patient management, personality assessments can aid driver safety screening and education. For instance, Sweden’s commercial driver licensing programmes use personality-based assessments to identify risky traits, enabling early intervention (36). Customised safety programmes focusing on extraversion’s link to injury proneness and openness protective effects can improve public safety efforts and inform policy development (21).

Incorporating personality assessments into routine medical evaluations, such as annual check-ups, could help identify individuals at a higher risk of injuries. Such assessments would allow healthcare providers to offer early, personalised guidance and preventive strategies, contributing to a proactive approach to injury prevention, as seen with extroverted athletes in New Zealand (37). This strategy could support broader public health efforts by addressing behavioural risk factors before injuries occur.

## Limitations

### Study Setting

This study was conducted exclusively in an ED, where acute emotional stress may have influenced patients’ responses to personality assessments, potentially affecting the accuracy of self-reported data (38).

### Cross-sectional Design

The cross-sectional nature of this study provides only a snapshot in time, limiting the ability to establish causality between personality traits and injury risk.

### Selection Bias

The sample comprised only individuals seeking medical attention for minor injuries, potentially excluding individuals with similar injuries who chose not to seek care. This may affect the generalisability of the findings, as non-attendees may exhibit different personality traits.

### Injury Severity Scope

This study focused solely on minor injuries, limiting the findings’ applicability to more severe cases.

### Self-reported Personality Data

Self-reported personality assessments introduce the potential for social desirability bias, as participants may respond in ways they view as socially acceptable.

## Recommendations for Future Research

While this study sheds light on the link between personality traits, gender, and injury proneness, further research is necessary to understand these complex relationships fully. To improve this study, a longitudinal design tracking patients from pre-injury to post-recovery would offer deeper insights into the causal relationships between personality traits and injury risk. Expanding the participant pool to include individuals seeking medical care in non-emergency settings and a spectrum of different severity of injuries would provide a more holistic understanding of how personality traits influence injury outcomes. Future studies should incorporate objective measures alongside self-reports, such as observational data, to enhance the validity of the results.

## Conclusion

In summary, this study highlights the role and influence of extraversion and openness on injury risk among minor injury patients at Hospital USM, with extraversion associated with a higher risk and openness providing a protective effect. Other traits, including neuroticism, conscientiousness, and agreeableness, were not significantly associated with injury risk. Further research is needed, given the study’s focus on minor injuries and the ED setting. Future studies should employ longitudinal designs to explore how personality traits influence injury risk over time and across a broader range of injury severities. Integrating personality assessments into injury prevention strategies could enhance patient care and support public health initiatives aimed at reducing injury risk through personalised interventions.

## Figures and Tables

**Table 1:** Demographic characteristics (*n* = 150)

Variable		No Injury		Injury		*P*-value*

		*n*	%	*n*	%	
Gender	Female	46	61.3	24	32.0	< 0.001
	Male	29	38.7	51	68.0	

Age (years)	18–29	47	62.7	44	58.7	0.362
	30–39	20	26.7	18	24.0	
	40–49	3	4.0	9	12.0	
	50–60	5	6.7	4	5.3	
Education	School	25	33.3	36	48.0	0.153
	Diploma	24	32.0	22	29.3	
	Degree/Master	26	34.7	17	22.7	

Employment	No	28	37.3	23	30.7	0.389
	Yes	47	62.7	52	69.3	

Location of accident	Work	0	0	14	18.7	N/A
	Home	0	0	9	12.0	
	Outdoor	0	0	52	69.3	

Notes: *n* = frequency; N/A = no statistics are computed because injury is a constant;

* chi-square test

**Table 2:** Comparison of personality traits between minorly injured patients and non-injury-related patients

Variable	No Injury		Injury		*P*-value*

	Mean	SD	Mean	SD	
Extraversion	2.29	0.42	2.46	0.53	0.033
Conscientiousness	2.62	0.48	2.68	0.51	0.457
Agreeableness	2.59	0.42	2.65	0.41	0.374
Neuroticism	1.76	0.50	1.61	0.60	0.094
Openness	2.46	0.45	2.28	0.52	0.023
Fake index	2.32	0.56	2.15	0.50	0.054

Note:

* independent *t*-test

**Table 3:** Factors associated with injury using SLR analysis (*n* = 150)

Variable		Crude OR	95% CI		*P*-value*

			Lower	Upper	
Gender	Female	ref.			
	Male	3.371	1.722	6.599	< 0.001

Age (years)	18–29	1.170	0.295	4.640	0.823
	30–39	1.125	0.261	4.848	0.874
	40–49	3.750	0.587	23.936	0.162
	50–60	ref.			

Education	School	2.202	0.993	4.883	0.052
	Diploma	1.402	0.604	3.253	0.431
	Degree/Master	ref.			

Employment	No	ref.			
	Yes	1.347	0.684	2.654	0.389

Extraversion		1.064	1.004	1.127	0.036

Conscientiousness		1.021	0.967	1.078	0.454

Agreeableness		1.030	0.965	1.100	0.372

Neuroticism		0.959	0.913	1.008	0.097

Openness		0.937	0.884	0.992	0.025

Notes: ref. = reference group;

* SLR

**Table 4:** Factors associated with injury using MLR analysis (*n* = 150)

Variable		Adjusted OR	95% CI		*P*-value*

			Upper	Lower	
Gender	Female	ref.			0.004
	Male	2.797	1.391	5.623	

Extraversion		1.092	1.020	1.169	0.012

Openness		0.916	0.856	0.980	0.011

Notes:

* MLR;

ref. = reference group; constant = −0.569; backward likelihood ratio (LR) were applied; no multicollinearity and no interaction; Hosmer Lemeshow test, *P*-value = 0.515; classification table 69.3% correctly classified; area under receiver operating characteristics (ROC) curve( was 73.0% *P* < 0.001)

**Table 5:** Comparison of personality traits between genders (*n* = 150)

Variable	Female		Male		*P*-value*

	Mean	SD	Mean	SD	
Extraversion	27.74	5.68	29.16	5.88	0.068
Conscientiousness	31.90	5.54	31.71	6.24	0.423
Agreeableness	31.79	4.99	31.11	4.91	0.204
Neuroticism	20.63	6.03	19.93	7.31	0.262
Openness	29.40	5.73	27.58	5.97	0.029
Fake Index	13.31	3.27	13.53	3.21	0.346

Note:

* independent *t*-test
